# Reply to Markaryan et al. Comment on “Aji et al. aMMP-8 POCT vs. Other Potential Biomarkers in Chair-Side Diagnostics and Treatment Monitoring of Severe Periodontitis. *Int. J. Mol. Sci.* 2024, *25*, 9421”

**DOI:** 10.3390/ijms26189074

**Published:** 2025-09-18

**Authors:** Nur Rahman Ahmad Seno Aji, Julie Toby Thomas, Nilminie Rathnayake, Fionnuala T. Lundy, Maelíosa T. C. Mc Crudden, Lata Goyal, Vaibhav Sahni, Miika T. Penttala, Andreas Grigoriadis, Tommi Pätilä, Pirjo Pärnänen, Dimitra Sakellari, Dirk Neefs, Andreas Pfützner, Shipra Gupta, Timo Sorsa, Ismo T. Räisänen

**Affiliations:** 1 Department of Oral and Maxillofacial Diseases, Head and Neck Center, University of Helsinki and Helsinki University Hospital, 00290 Helsinki, Finland; 2 Department of Periodontics, Faculty of Dentistry, Universitas Gadjah Mada, Jalan Denta No. 1 Sekip Utara, 10 Sleman, Yogyakarta 55281, Indonesia; 3 Wellcome-Wolfson Institute for Experimental Medicine, School of Medicine, Dentistry and Biomedical Science, Queen’s University Belfast, Belfast BT9 7BL, UK; 4 Periodontics Division, Department of Dentistry, All India Institute of Medical Sciences, Bathinda 151001, India; 5 Research & Evidence (RF&E), New Delhi 110001, India; 6 Department of Preventive Dentistry, Periodontology and Implant Biology, Dental School, Aristotle University of Thessaloniki, 54124 Thessaloniki, Greece; 7 Dental Sector, 424 General Military Training Hospital, 56429 Thessaloniki, Greece; 8 Department of Congenital Heart Surgery and Organ Transplantation, New Children’s Hospital, University of Helsinki, 00290 Helsinki, Finland; 9 Department of Biomedical Surgical and Dental Sciences, Faculty of Medicine and Surgery, University of Milan, 20122 Milan, Italy; 10 Department of Internal Medicine and Laboratory Medicine, University for Digital Technologies in Medicine and Dentistry, 9516 Wiltz, Luxembourg; 11 Oral Health Sciences Centre, Post Graduate Institute of Medical Education & Research, Chandigarh 160012, India; 12 Division of Oral Diseases, Department of Dental Medicine, Karolinska Institute, 17177 Stockholm, Sweden

## 1. Introduction

**Figure 1:**
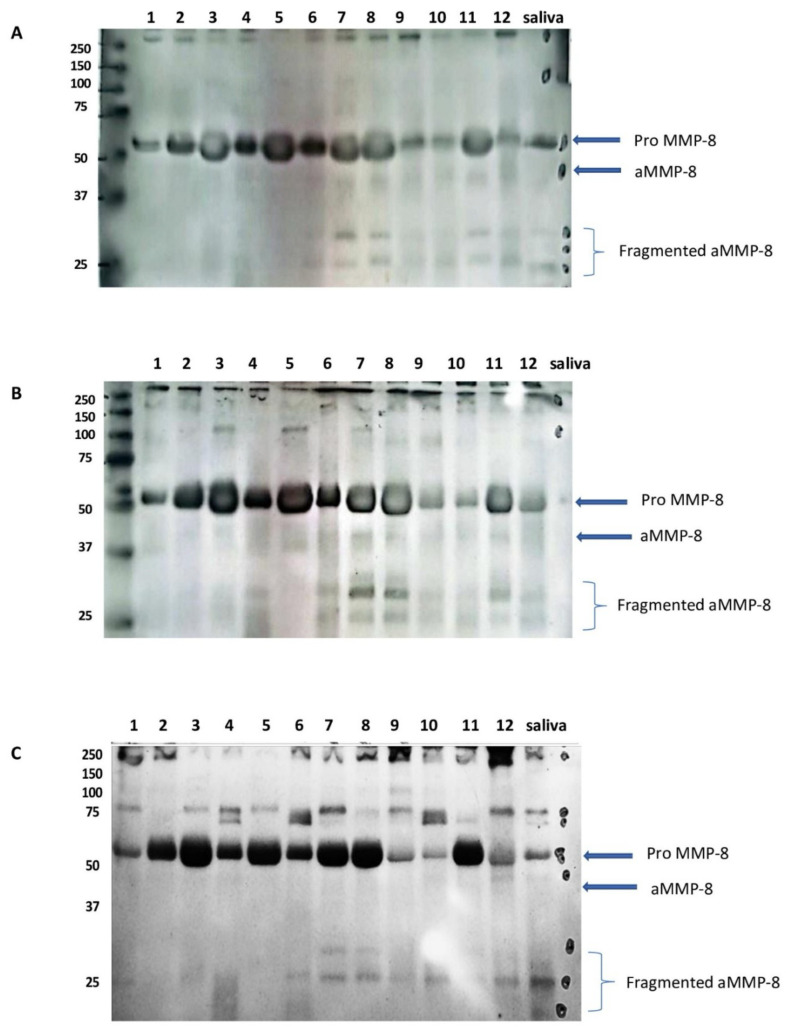
(**A**,**B**) Western immunoblot analysis using MMP-8 monoclonal antibodies; (**C**) Western blot analysis used polyclonal antibodies. This figure shows that healthy samples (lanes 1–6) show intact pro-latent total collagenase (pro MMP-8), and periodontitis stage III/IV-grade B/C samples (lanes 7–12) reveal activated collagenase (aMMP-8) that concomitantly also fragmented to lower molecular species, without exception. Healthy control saliva is a positive control (lane saliva). Molecular weight markers’ mobilities are shown on the left. This was an observational study.

We appreciate Markaryan et al. [1] for raising timely and important queries on the role of aMMP-8 (active-matrix metalloproteinase-8) and tMMP-8 (total latent 75 and 55 kDa proMMP-8) in periodontal diagnostics and our ability to compare them. MMP-8 in periodontitis- and peri-implantitis-affected gingiva, gingival crevicular fluid (GCF) and peri-implant sulcular fluid (PISF), mouth rinse, and saliva is converted to its 10 kDa lower-molecular-size active forms and, at the same time, further fragmented (Figure 1A–C), whereas the MMP-8 in the oral fluids of healthy gingival tissues, gingivitis, and mucositis exists mostly as the latent or total MMP-8 pro-form. The same MMP-8 activation of proMMP-8 has been demonstrated in wound healing in patients who have undergone, for example, excimer laser photorefractive keratectomy [2,3]. aMMP-8 is a catalytically competent collagenolytic and proteolytic immunomodulator [4,5,6]. In contrast, the latent, total, or pro 75 and 55 kDa MMP-8 forms are catalytically inactive, lacking collagenolytic and proteolytic activity. This fundamental distinction has been rigorously demonstrated through various collagen degradation diagnostic assays conducted before the advent of the antibody-based enzyme-linked immunosorbent assay (ELISA), the time-resolved immunofluorescence assay (IFMA), and chair-side immunoassay techniques [7,8,9,10].

The role of aMMP-8 as a key biomarker in periodontitis and its diagnostic potential have been extensively illustrated in previous research [11,12,13,14,15]. Studies using collagen degradation activity assays have provided strong evidence supporting this conjecture [7,8,9,10]. These foundational studies form the scientific basis for subsequent research and development and support the necessity of anti-MMP-8 polyclonal and monoclonal antibodies, as well as the development of both immunoassays and chair-side assays. Key studies that have contributed to this field include those by Sorsa et al. [16] and Mäntylä et al. [17], both of which utilized and successfully validated a diagnostic cut-off value of 20 ng/mL for aMMP-8 detection. Penttala et al. [18] recently had success in utilizing an aMMP-8 mouth rinse cut-off value of 20 ng/mL in an algorithmic mobile diagnostic procedure capable of delivering results in 5 min and 1s. AI applications [18] would not have been possible with cut-offs at 10 ng/mL [19] and 25 ng/mL [20,21].

Nonetheless, variations in cut-off values have been reported in some studies. For instance, Deng et al. [19] have explored lower thresholds such as 10 ng/mL, while Lorenz et al. [20] have used 25 ng/mL. Despite these variations, independent studies conducted in India, Sweden, Greece, Finland, Germany, Holland, and Nigeria, by researchers including Aji et al. [22], have consistently validated the 20 ng/mL cut-off as an effective and optimal threshold for periodontitis risk assessment. Notably, Aji et al. [23] demonstrated that cut-offs of 10 ng/mL and 25 ng/mL do not provide reliable diagnostic accuracy. These findings underscore the robustness of the 20 ng/mL cut-off, reinforcing its clinical reliability and widespread adoption in non-invasive periodontal screening methodologies.

## 2. Comments and Discussion

In response to the first point by Markaryan et al. [1], the monoclonal antibody utilized in the aMMP-8 point-of-care test (POCT) PerioSafe/Oralyzer and IFMA is the same as the one described in Sorsa et al.’s US patents. This antibody is designed to detect active MMP-8, its activation products, and related lower-molecular-weight fragments at the same time (Figure 1A–C). Independent studies have also confirmed these findings using both monoclonal and polyclonal anti-MMP-8 antibodies [9,17]. While Lee et al. [7] did not investigate antibodies but mainly collagen degradation assay in their research, Romanelli et al. [9] provided further validation of these results through independent monoclonal antibody studies. Additionally, activation-related lower-molecular-size MMP-8 fragments (Figure 1A–C) are believed to not be collagenolytic, as shown by Hasty et al. [24] and Knäuper et al. [25], but formed from aMMP-8 during activation. Periodontitis- or kidney disease-related activation with concomitant further fragmentation of active MMPs (Figure 1A–C) is not only a characteristic of MMP-8 but also concerns MMP-9, -13, and -14 [9,24,25].

In response to the second, third, and fourth points, our findings indicate that tMMP-8 ELISAs are not effective in diagnosing periodontitis or serving as a reliable biomarker within the new 2017 classification system [26,27,28,29], which is related to a significant extent to the work by Sorsa et al. in terms of utilizing aMMP-8 as a biomarker for this system for periodontal or peri-implant disease [22]. Instead, clinicians and researchers should prioritize methods that specifically target aMMP-8, as it provides a more precise reflection of ongoing collagen breakdown, inflammation, and clinically progressive soft and hard tissue destruction associated with periodontal and peri-implant diseases [7,8,9,10,15,23,30,31,32,33,34,35].

We compared the aMMP-8 POCT Oralyzer chair-side immunotest with the catalytic active collagenase activity assay [22]. The Academic Editor’s comment on this comparison is both relevant and valuable. In response, we emphasize the following: even if the MMP-8 antibody can detect both active and inactive MMP-8, the detection method (FRET substrate) enables only the measurement of the active form of MMP-8 [22]. The FRET substrate was not claimed to be specific to MMP-8. The specificity of the assay comes from the use of the capture antibody [22]. Our objective was to compare POCT with an entirely independent catalytic activity assay, which would not have been feasible if the same antibody had been used in both methods [22].

Furthermore, multiple international and independent cohort studies have consistently shown that aMMP-8 POCT and IFMA outperform total MMP-8 ELISA in diagnostic precision. Across all assays, aMMP-8 POCT and IFMA have demonstrated superior diagnostic accuracy in relation to tMMP-8 ELISAs [16,22]. These studies, conducted across diverse international populations or cohorts and clinical settings, reinforce the clinical reliability and accuracy of aMMP-8-based assays in detecting active periodontal peri-implant destruction.

**Figure 2:**
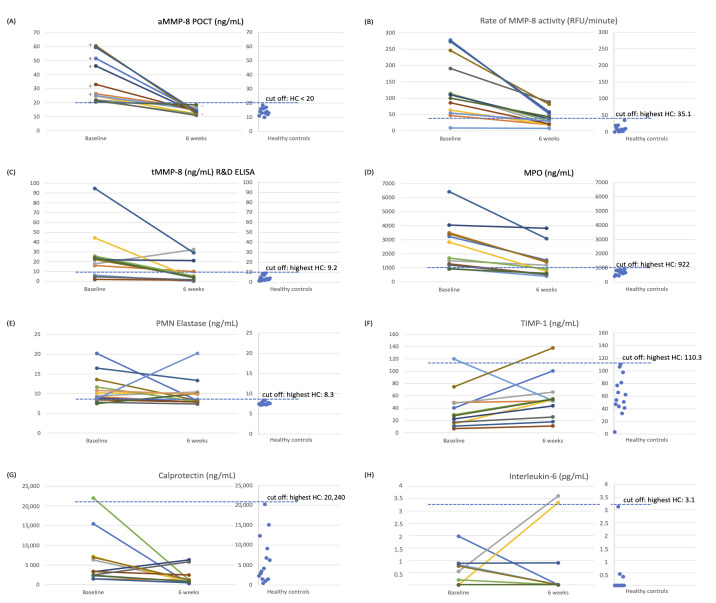
Treatment effect monitoring by oral fluid biomarkers: In the figure, the various colors correspond to different individual patient samples. (**A**) aMMP-8 POCT (ng/mL) (PerioSafe/Oralyzer) with cut-off 20 ng/mL (test positive, +, ≥20 ng/mL; test negative, −, <20 ng/mL), (**B**) rate of MMP-8 activity (RFU/minute), (**C**) tMMP-8 (ng/mL) (R&D systems ELISA), (**D**) MPO (ng/mL), (**E**) PMN Elastase (ng/mL), (**F**) TIMP-1 (ng/mL), (**G**) Calprotectin (ng/mL), and (**H**) Interleukin-6 (ng/mL) compared to the highest value of the healthy controls (3rd-year dental students (HCs). According to Aji et al. [22], 13 adult patients with chronic stage III/IV grade B/C periodontitis were treated by non-surgical periodontal treatment and were compared to 13 systematically and periodontally healthy controls (HCs) [22]. This was an observational study.

In the present study [22], full-mouth clinical parameters and biomarkers, i.e., the aMMP-8 POCT, the aMMP-8 RFU activity [22], total MMP-8 ELISA, myeloperoxidase, PMN elastase, TIMP-1, calprotectin, and interleukin-6, were analyzed and compared at baseline and after non-surgical therapy at 6 weeks (Figure 2). The aMMP-8 POCT with a cut-off at 20 ng/mL, but not with cut-offs of 10 ng/mL and 25 ng/mL, and myeloperoxidase were the most efficient in discriminating between periodontal health and disease (measured by ROC AUC [22]) followed closely by aMMP-8 RFU activity and PMN elastase. In comparison, total MMP-8 R&D ELISA was found a little less efficient in that regard, while TIMP-1, calprotectin, and interleukin-6 were the least precise. Similar findings were found in the treatment effect monitoring ([23] and Figure 2). Additionally, GCF samples from six healthy and six periodontitis-affected sites (Stage III/IV, Grade B/C) were analyzed via Western blotting for MMP-8 using a polyclonal anti-MMP-8 and monoclonal anti-MMP-8 antibodies (Figure 1A–C). Western blotting results clearly show that in healthy GCF, MMP-8 is predominantly in its latent (total) pro-form. However, in periodontitis-affected GCF, MMP-8 is consistently and without exception converted to its active form, with the concomitant formation of lower-molecular-weight fragments, as confirmed by both polyclonal and monoclonal antibodies (Figure 1A–C). Finally, there is the dento-ELISA with Sorsa et al.’s US patent-19 anti-aMMP-8-monoclonal antibodies, as well [16]. Thus, targeting these MMP-8 activation species can be achieved conveniently by multiple diagnostic methods utilizing Sorsa et al.’s US patent-19 antibodies in oral fluids and human excretion fluids [3,16].

## 3. Conclusions

**Figure 3:**
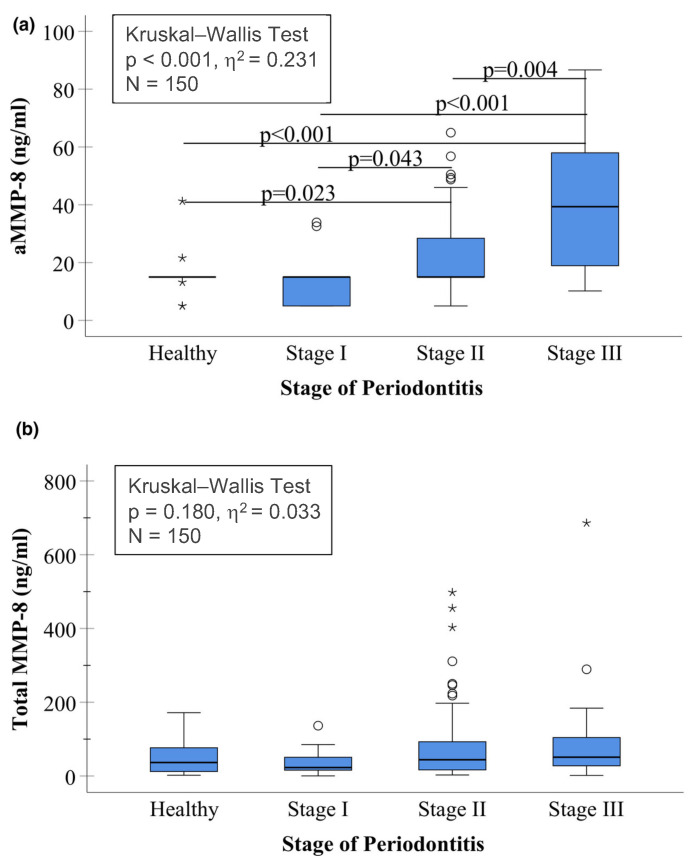
Box plot of concentrations of (**a**) active MMP-8 (aMMP-8) measured by quantitative lateral flow mouth rinse point-of-care technology (POCT) (PerioSafe/ORALyzer combination) and (**b**) total (latent and active) salivary MMP-8 measured by ELISA, Quantikine, R&D Systems, categorized by stage of periodontitis in 150 Greek adults, as described previously [35]. The asterisk (*) indicates outliers of more than 3 times the interquartile range and the circle (○) indicates outliers between 1.5–3 times the interquartile range in the data. This figure is reproduced under the terms and conditions of the Creative Commons Attribution (CC BY) license https://creativecommons.org/licenses/by/4.0/ (accessed on 4 September 2025) [35].

These findings clearly indicate that active MMP-8, together with its lower-molecular-weight fragments, serves as a biomarker of MMP-8 activation in periodontitis (Figure 1 and Figure 2), further supported by the independent international studies [23]. Previous studies have shown that collagenolytic aMMP-8 with a cut-off at 20 ng/mL—but not the non-collagenolytic total MMP-8—can be implemented into the 2017 periodontitis classification system as a biomarker of the staging and grading of periodontitis (Figure 3) [23,29]. Thus, we should not synonymize aMMP-8 and total MMP-8 in periodontitis diagnostics. The aMMP-8-test technology is commercially available as IFMA, dento-ELISA, RFU, and POCT-Oralyzer chair-side assays [16,22,23].

## Data Availability

Data supporting reported the results can be obtained from the authors on request.
